# Malnutrition and nutrition impact symptoms (NIS) in surgical patients with cancer

**DOI:** 10.1371/journal.pone.0241305

**Published:** 2020-12-15

**Authors:** Eduarda Cristina Rodrigues de Morais Viana, Isadora da Silva Oliveira, Ana Beatriz Rechinelli, Isabele Lessa Marques, Vanusa Felício de Souza, Maria Cláudia Bernardes Spexoto, Taísa Sabrina Silva Pereira, Valdete Regina Guandalini

**Affiliations:** 1 Department of Integrated Education, Federal University of Espirito Santo, Vitoria, ES, Brazil; 2 Faculty of Pharmaceutical Sciences, Nutrition Course, Federal University of Grande Dourados, Dourados, Mato Grosso do Sul, Brazil; 3 Universidad de las Américas Puebla, Cholula, Puebla, México; 4 Postgraduate Program in Nutrition and Health, Federal University of Espirito Santo, Vitoria, ES, Brazil; University of Agriculture in Krakow, POLAND

## Abstract

**Background and aim:**

Nutrition impact symptoms (NIS) are common in cancer patients and the negative impacts on nutritional status indicate the need for research, diagnosis and nutritional intervention in order to reduce the risk of malnutrition. We aimed to verify the presence of malnutrition, the need for nutritional intervention, NIS and their influence on the nutritional status of surgical patients with cancer.

**Methods:**

This cross-sectional study was carried out in a public tertiary hospital, from March 2017 to October 2019. Nutritional status, the need for nutritional intervention and NIS were assessed through the Patient-Generated Subjective Global Assessment (PG-SGA) in the first 48 hours of hospital admission.

**Results:**

Among the 135 patients evaluated, 55.6% were elderly and 51.1% were male; patients had a median age of 62 years and a predominance of cancer located in the lower gastrointestinal tract (35.6%). Malnutrition and the need for nutritional intervention were identified in 60.0% and 90.4% of cases, respectively. The presence of three or more NIS was reported by 51.9% of patients. Significant differences in NIS were observed according to sex, PG-SGA classification and PG-SGA score. After logistic regression analysis, it was determined that the symptoms that increased the chances of malnutrition were anorexia, constipation, strange taste, mouth sores and others (depression, dental or financial problems).

**Conclusion:**

Malnutrition, the need for nutritional intervention and the presence of three or more NIS were elevated in the patients evaluated. Malnutrition was associated with the presence of NIS, indicating the need for attention and care in antineoplastic treatment.

## Introduction

Cancer is the second leading cause of death in the world and was responsible for 9.6 million deaths in 2018 [1,2]. Malnutrition is one of the main complications in these patients, with negative impacts on quality of life, reduced immune response, tolerance to antineoplastic treatment and survival, in addition to increasing postoperative complications, hospital stay and costs [3–5].

The prevalence of malnutrition is around 40% to 80% in cancer patients [6]. In a study carried out in all regions of Brazil on hospitalized patients with cancer using the Patient-Generated Subjective Global Assessment (PG-SGA), it was found that 45.3% of the patients had some degree of malnutrition (moderate or severe) [5].

Among the prognostic indicators of specific malnutrition for cancer patients, we highlight the presence of nutrition impact symptoms (NIS) which assess the impediments to adequate food intake that can occur, such as anorexia, unwillingness to eat, nausea, constipation, mouth sores, pain and vomiting, mainly due to factors related to the tumor itself, such as location, staging and type of treatment [7].

NIS are common in cancer patients and studies in the literature have investigated their impact on nutritional status [4,5,8]. In a study carried out on 52 patients with advanced cancer, the most frequent symptoms were changes in taste and smell, constipation, abdominal pain, dysphagia and epigastric pain [3].

In a multicenter study, carried out on 4783 cancer patients, it was observed that the presence of three or more NIS was associated with malnutrition in 51.8% of malnourished patients. The symptoms that most influenced nutritional status were difficulty in swallowing, loss of appetite, vomiting, early satiety, diarrhea and nausea. In addition, almost half of the patients needed nutritional intervention and symptom management [5].

Similar results were observed by Galindo et al. [9]; in their study, changes in appetite were associated with malnutrition and a worse prognosis by PG-SGA and anthropometric parameters. The combination of anorexia and early satiety was associated with lower current weight and body mass index (BMI). Appetite disorders are highly prevalent among cancer patients at risk of malnutrition or malnutrition, and have a significant impact on nutritional status, especially when anorexia and early satiety are combined.

Thus, when using PG-SGA as the preferred tool for nutritional diagnosis, it is also possible to verify the presence of NIS and their impact on nutritional status and the need for nutritional intervention, since their management can prevent malnutrition and provide individualized and early clinical and nutritional care.

Based on this hypothesis, this study aimed to verify the presence of malnutrition, the need for nutritional intervention, NIS and their influence on the nutritional status of surgical patients with cancer.

## Methods

### Subjects and study period

This descriptive cross-sectional study was carried out in a public tertiary hospital–a referral center for gastrointestinal tract surgery–located in Vitória-ES, Brazil, from March 2017 to October 2019.

### Study population

The study included patients of both sexes, aged 20 years or over, evaluated within the first 48 hours of hospital admission, with a confirmed diagnosis of cancer, admitted to the General Surgery Unit to perform a curative surgical. Individuals with cognitive dysfunction predicted in medical records, in respiratory isolation, in palliative care or with whole body edema were excluded.

Tumor location was grouped into the upper gastrointestinal tract (GIT), lower GIT, adnexal glands (liver, pancreas and bile ducts) and others (unknown behavior, skin cancer, ovary, thyroid, peritoneum, mediastinum, pleura, cervical, submandibular and connective tissue, lung, skin and hematological cancer).

Patients were grouped into adults (<60 years old) and elderly (≥ 60 years old), according to the WHO classification for developing countries [10].

### Study variables

Specific protocols were applied in order to collect sociodemographic (age, sex, race/self-declared color) and anthropometric (weight (kg), height (m)) information, according to standardized protocols [11,12]. After this stage, PG-SGA was applied.

### Patient-Generated Subjective Global Assessment (PG-SGA)

For this study, the version of PG-SGA translated and validated for Brazilian Portuguese by Gonzalez et al. [13] was used, with permission to use PG-SGA/Pt-Global Platform (www.pt-global.org). PG-SGA is recommended by the Brazilian Consensus of Oncological Nutrition for the evaluation of cancer patients in Brazil [14].

The version used is divided into two parts. The first part evaluates issues related to nutritional risk symptoms, common in cancer patients, such as changes in weight, changes in food intake and NIS, and activity and function. The second part assesses issues based on factors associated with the presence of metabolic stress (fever and use of corticosteroids), depletion of physical status (changes in fat reserves, muscle mass and water retention), percentage weight loss in 1 month or 6 months, and the presence of other factors related to health conditions such as cancer, pulmonary or cardiac cachexia, decubitus ulcer, presence of trauma, age over 65 years and acquired immunodeficiency syndrome (SIDA) (boxes A–D). In this study, the evaluators helped to read and understand the questionnaire.

The NIS are part of box 3 and the frequency with which symptoms appeared was observed; these include: no problems with food, no appetite, just no desire to eat, nausea (feeling sick), constipation (stuck intestine), mouth sores, things taste strange or have no taste, smells bother me, I feel quickly satisfied, pain, vomiting, diarrhea, mouth dryness, problems with swallowing, tiredness (fatigue) and others, for example depression and dental or financial problems.

PG-SGA classifies nutritional status into three categories: well nourished (A), suspected or moderately malnourished (B) and/or severely malnourished (C). This instrument also allows assessment of the need for nutritional intervention through the sum of the scores at four levels: 0–1 points: no intervention needed at the moment. Reassess routinely and regularly during treatment; 2–3 points: counseling of the patient and his or her family by a nutritionist, nurse or other clinician, with pharmacological intervention as indicated by the evaluation of symptoms and laboratory tests, as appropriate; 4–8 points: requires intervention by a nutritionist, together with a nurse or doctor as indicated by the symptoms; and ≥ 9 points: indicates an urgent need for conduct to improve symptoms and/or nutritional intervention options.

### Ethical aspects

The study was conducted in accordance with Resolution 466 of December 08, 2012, of the National Health Council of Brazil. Ethical approval for this study was obtained from the Research Ethics Committee of the Federal University of Espirito Santo–Protocol 2.141.932/2017. Patients participated voluntarily and provided written informed consent.

### Statistical analysis

A descriptive analysis was performed expressed in means and standard deviations to describe the continuous and percentage variables for the categorical variables. The Kolmogorov–Smirnov test was used to verify the normality of quantitative variables. Age and PG-SGA score did not present a normal distribution. Global classification of PG-SGA was grouped into three categories (well nourished (A), suspected of malnutrition or moderately malnourished (B) and severely malnourished (C)) and its score into: no need for nutritional intervention (0–3 points), need for nutritional intervention and symptom improvement (≥ 4 points). Fisher’s exact and chi-square tests were applied to verify the difference between proportions. For data analysis, race/color was grouped into whites and non-whites. Multivariate logistic regression models were applied considering two possible outcomes: moderate malnutrition and severe malnutrition. All variables that presented p values < 0.20 in the bivariate associations were included in the modeling. Those with p values < 0.05 were maintained, for which odds ratios and their respective confidence intervals were calculated. Data were analyzed with SPSS 21.0 software. A 5.0% significance level was used for all tests.

## Results

One hundred and thirty-five patients were evaluated, with a median age of 62 years. Of these, 55.6% (n = 75) were elderly, 51.1% (n = 69) male and 60.7% (n = 82) not white. Cancer of the lower GIT was prevalent, affecting 35.6% (n = 48) of those evaluated (Table 1).

**Table 1:** Demographic and clinical characteristics of cancer patients and hospitalized.

Variables	n (%)
**Age** (median)	62.0 years
**Min–Max.**	20–88 years
**Stage of life**	**n (%)**
Adult	60 (44.4)
Elderly	75 (55.6)
**Sex**	
Male	69 (51.1)
Female	66 (48.9)
**Ethnicity**	
White	53 (39.3)
Non-White	82 (60.7)
**Tumor location**	
Lower gastrointestinal tract	48 (35.6)
Adnexal glands	35 (25.9)
Upper gastrointestinal tract	23 (17.0)
Other*	29 (21.5)

GI: Gastrointestinal tract;

*Other: 25.0% for unknown behavior; 18.7% for cancer of the skin; 12.5% for ovarian cancer; 12.5% for thyroid cancer; 6.25% for peritoneum cancer; 6.25% for mediastinum cancer; 6.25% for pleura cancer; 6.25% for cervical cancer; 6.25% for submandibular cancer; 6.25% for connective tissue cancer. 5.9% for lung cancer. 3.7% for hematological cancer.

Table 2 shows the nutritional status, need for nutritional intervention, number of NIS and percentage weight loss (% WL) in the last 6 months. According to PG-SGA, 37.8% (n = 51) of patients were suspected or moderately malnourished and 22.2% (n = 30) were severely malnourished. The PG-SGA score indicated 90.4% of patients in need of nutritional intervention and improvement of symptoms. There was a higher proportion of individuals (51.8%; n = 70) with three or more NIS.

**Table 2:** Nutritional status, need for nutritional intervention, number of nutrition impact symptoms and percentage weight loss in cancer patients and hospitalized.

Variables	n (%)
**PG-SGA**	
Well nourished (A)	54 (40.0)
Suspected or moderately malnourished (B)	51 (37.8)
Severely malnourished (C)	30 (22.2)
**Nutritional intervention**	
Without need	13 (9.6)
With need	122 (90.4)
**%Weight loss 6 months**	
Without loss	51 (37.8)
<10.0%	63 (46.7)
≥ 10.0%	21 (15.6)
**Number of NIS**	
Whitout symptoms	12 (8.9)
< 3	53 (39.3)
≥ 3	70 (51.8)

PG-SGA: Patient-Generated Subjective Global Assessment; NIS: Nutrition impact symptoms.

Table 3 shows the presence of NIS according to sociodemographic variables, tumor location, nutritional status and need for nutritional intervention. Significant differences were observed between NIS and sex (p = 0.048), PG-SGA (p < 0.001) and the need for nutritional intervention (p < 0.001).

**Table 3:** Presence of NIS according to sex, stage of life, ethnicity, tumor location, nutritional status, weight loss and need for nutritional intervention in cancer patients and hospitalized.

Variables	Whitout symptoms	< 3 NIS	≥ 3 NIS	p value
**Sex**^a^				0.048*
Male	10 (14.5)	23 (33.3)	36 (52.2)	
Female	2 (3.0)	20 (30.3)	44 (66.7)	
**Stage of life**^a^				0.263
Adult	3 (5.0)	22 (36.7)	35 (58.3)	
Elderly	9 (12.0)	21 (28.0)	45 (60.0)	
**Ethnicity**^b^				0.349
Non-White	5 (6.1)	28 (34.1)	49 (59.8)	
White	7 (13.2)	15 (28.3)	31 (58.5)	
**Tumor location**^b^				0.213
Upper GI	1 (4.30)	8 (34.8)	14 (60.9)	
Lower GI	4 (8.3)	20 (41.7)	24 (50.0)	
Adnexal glands	5 (14.3)	5 (14.3)	25 (71.4)	
Other	2 (6.9)	10 (34.5)	17 (58.6)	
**PG-SGA**^a^				<0.001*
Well nourished	11 (20.4)	28 (51.9)	15 (27.8)	
Malnourished (B + C)	1 (20.4)	15 (18.5)	65 (80.2)	
**Nutritional intervention**				<0.001*
Without need	6 (46.2)	7 (53.8)	-	
With need	6 (4.9)	36 (29.5)	80 (59.3)	
**%Weight loss 6 months**^b^				0.385
Without loss	6 (11.8)	19 (37.3)	26 (51.0)	
< 10.0%	6 (9.5)	18 (28.6)	39 (61.9)	
≥ 10.0%	-	6 (28.6)	15 (71.4)	

^a^Qui-square test;

^b^Fisher's exact test. PG-SGA: Patient-Generated Subjective Global Assessment.

The associations between nutritional status and NIS are shown in Table 4. Nutritional status was associated with anorexia (p = 0.002), nausea (p < 0.001), constipation (p < 0.001), mouth sores (p = 0.001), strange taste (p < 0.001), uncomfortable odors (p = 0.011), vomiting (p < 0.001), dry mouth (p = 0.001), problems with swallowing (p = 0.004) and pain (p = 0.010).

**Table 4:** Association of nutritional status with nutrition impact symptoms.

PG-SGA					
NIS	Total n (%)	A	B	C	p value
**No appetite**^a^					0.002
Yes	47 (34.8)	10 (18.5)	26 (51)	11 (36.7)	
No	88 (65.2)	44 (81.5)	25 (49)	19 (63.3)	
**Nausea**^a^					<0.001
Yes	49 (36.3)	10 (18.5)	21 (41.2)	18 (60)	
No	86 (63.7)	44 (81.5)	30 (58.8)	12 (40)	
**Constipation**^a^					<0.001
Yes	44 (32.6)	6 (11.1)	25 (49)	13 (43.3)	
No	91 (67.4)	48 (88.9)	26 (51)	17 (56.7)	
**Mouth sores**^b^					0.001
Yes	17 (12.6)	1 (1.9)	8 (15.6)	8 (26.7)	
No	118 (87.4)	53 (98.1)	43 (84.3)	22 (73.3)	
**Strange taste**^a^					<0.001
Yes	38 (28.1)	5 (9.3)	21 (41.2)	12 (40.0)	
No	97 (71.9)	49 (90.7)	30 (58.8)	18 (60.0)	
**Smell bother**^a^					0.011
Yes	42 (31.1)	10 (18.5)	17 (33.3)	15 (50)	
No	93 (68.9)	44 (81.5)	34 (66.7)	15 (50)	
**Feel full quickly**^a^					0.180
Yes	55 (40.7)	17 (31.5)	23 (45.1)	15 (50)	
No	80 (59.3)	37 (68.5)	28 (54.9)	15 (50)	
**Vomiting**^a^					<0.001
Yes	34 (25.2)	6 (11.1)	11 (21.6)	17 (56.7)	
No	101 (74.8)	48 (88.9)	40 (78.4)	13 (43.3)	
**Diarrhea**^b^					0.487
Yes	21 (15.6)	6 (11.1)	10 (19.6)	5 (16.7)	
No	114 (84.4)	48 (88.9)	41 (80.4)	25 (83.3)	
**Dry mouth**					0.001
Yes	56 (41.5)	12 (22.2)	26 (51)	18 (60)	
No	79 (58.5)	42 (77.8)	25 (49)	12 (40)	
**Swallowing problem**^b^					0.004
Yes	21 (15.6)	3 (5.6)	8 (15.7)	10 (33.3)	
No	114 (84.4)	51 (94.4)	43 (84.3)	20 (66.7)	
**Pain**^a^					0.010
Yes	69 (51.1)	19 (35.2)	32 (62.7)	18 (60)	
No	66 (48.9)	35 (64.8)	19 (37.3)	12 (40)	
**Other**^a^**					0.219
Yes	31 (23)	9 (16.7)	12 (23.5)	10 (33.3)	
No	104 (77)	45 (83.3)	39 (76.5)	20 (66.7)	

^a^Qui-square test;

^b^Fisher's exact test. PG-SGA: Patient-Generated Subjective Global Assessment. NIS: Nutrition impact symptoms.

**Others: Depression and dental or financial problems.

Table 5 shows the gross and adjusted odds ratio (OR). We observed that the symptoms that presented a greater chance of leading to moderate malnutrition were anorexia (OR 5.72, 95% CI 1.49–21.99), constipation (OR 14.64, 95% CI 3.80–56.38) and strange taste (6.27, 95% CI 1.26–31.13), while the symptoms associated with severe malnutrition were constipation (OR 10.04, 95% CI 2.21–45.53), mouth sores (OR 13.87, 95% CI 1.09–176.42) and others (OR 5.19, 95% CI 1.10–24.54).

**Table 5:** Multivariate logistic regression according to the degree of malnutrition and the influence of nutrition impact symptoms.

	PG-SGA			
NIS	Moderately malnourished		Severely malnourished	
	OR Crude	OR Adjusted*	OR Crude	OR Adjusted*
**No appetite**				
Yes	**4.58 (1.90–11.02)**	**5.72 (1.49–21.99)**	2.55 (0.93–7.00)	1.46 (0.29–7.42)
No	1	1	1	1
**Nausea**				
Yes	**3.08 (1.27–7.46)**	1.05 (0.21–5.22)	**6.60 (2.42–17.98**)	2.15 (0.35–13.10)
No	1	1	1	1
**Constipation**				
Yes	**7.69 (2.80–21.14)**	**14.64 (3.80–56.38)**	**6.12 (2.01–18.64)**	**10.04 (2.21–45.53)**
No	1	1	1	1
**Mouth sores**				
Yes	**9.86 (1.19–81.94)**	6.94 (0.54–88.99)	**19.27 (2.27–163.39)**	**13.87 (1.09–176.42)**
No	1	1	1	1
**Strange taste**				
Yes	**6.86 (2.34–20.12)**	**6.27 (1.26–31.13)**	**6.53 (2.01–21.15)**	1.94 (0.31–11.98)
No	1	1	1	1
**Smell bother**				
Yes	2.20 (0.89–5.41)	0.35 (0.77–1.60)	**4.40 (1.63–11.86)**	0.92 (0.18–4.72)
No	1	1	1	1
**Feel full quickly**				
Yes	1.79 (0.81–3.96)	1.08 (0.31–3.71)	2.18 (0.87–5.45)	1.59 (0.39–6.38)
No	1	1	1	1
**Vomiting**				
Yes	2.20 (0.75–6.45)	0.31 (0.50–1.90)	**10.46 (3.43–31.88)**	2.21 (0.33–14.65)
No	1	1	1	1
**Diarrhea**				
Yes	1.95 (0.65–5.83)	1.85 (0.44–7.80)	1.60 (0.44–5.76)	0.78 (0.14–4.43)
No	1	1	1	1
**Dry mouth**				
Yes	**3.64 (1.56–8.47)**	4.26 (1.06–17.11)	**5.25 (1.98–13.88)**	2.88 (0.59–14.00)
No	1	1	1	1
**Swallowing problem**				
Yes	**3.16 (0.79–12.67)**	2.26 (0.34–14.75)	**8.5 (2.12–34.12)**	6.14 (0.95–39.60)
No	1	1	1	1
**Pain**^**a**^				
Yes	**3.10 (1.40–6.88)**	3.29 (0.92–11.70)	**2.76 (1.10–6.93)**	1.58 (0.35–7.01)
No	1	1	1	1
**Others**				
Yes	1.54 (0.59–4.03)	3.77 (0.94–15.16)	2.5 (0.88–7.01)	**5.19 (1.10–24.54)**
No	1	1	1	1

OR: Odds Ratio. Adjusted between them for sex and age.

p<0,05*.

## Discussion

Our results showed a high prevalence of malnutrition, the need for nutritional intervention and the presence of three or more NIS. In a multicenter study conducted in Brazil, 4783 cancer patients were evaluated using PG-SGA. Of these, 33.5% were suspected or moderately malnourished and 11.8% were severely malnourished [5].

The prevalence of malnutrition is one of the main characteristics of this population and is confirmed by different studies. Even though found in different proportions, with variations from 28.3% to 76.0% [15–17], it is a present condition that implies clinical, metabolic and nutritional problems and complications with a negative impact on quality of life, response to treatment and survival [18].

The need for nutritional intervention was high in this study. Vale et al. [19] pointed out that 66.0% of patients have a need for nutritional intervention at the beginning of treatment according to the numerical PG-SGA score and, of these, 44.0% have a critical need for intervention, results that are also confirmed for similar works [5,20,21].

The need for nutritional intervention is an indicator of worsening nutritional status, represented in this population by rapid weight loss, reduced food intake and functional capacity, in addition to the presence of NIS, and directs care and nutritional conduct according to their gravity.

The significant presence of NIS in this study was associated with malnutrition and the critical need for nutritional intervention, as previously described in the literature [3–5]. NIS such as changes in taste and smell can be silent and frequent, as they are not always reported by patients or evaluated by health professionals [3].

Pinho et al. [5], when assessing the relationship between malnutrition and NIS, demonstrated that patients with three or more NIS were more malnourished. The presence of three or more NIS was observed in 34.0% of patients with moderate malnutrition and in 51.8% of patients with severe malnutrition.

The patient’s diet is influenced by the disease, treatment, and psychological and emotional factors [22]. In the case of malnutrition, one of the main causes is inadequate food consumption due to changes in appetite and difficulty in eating food [23]. Inadequate food consumption can lead to metabolic complications and other serious manifestations that can increase morbidity, mortality and worsen the response to treatment [24].

In our study, the NIS associated with the nutritional status observed were anorexia, nausea, constipation, mouth sores, strange taste, vomiting, dry mouth, problems with swallowing and pain.

Omlin et al. [3] evaluated the NIS in cancer patients and the most frequent ones were changes in taste and smell, constipation, abdominal pain, dysphagia and epigastric pain. NIS have been shown to be constant in these patients and, therefore, attention should be given to them by the multidisciplinary team, since they are directly associated with malnutrition. Still neglected, NIS should be integrated into the screening protocol and initial nutritional assessment.

NIS were evaluated in a recent cross-sectional study with 133 patients with chronic liver disease undergoing liver transplantation. Even using instruments other than PG-SGA, the NIS that were more frequent, regardless of severity, were pain, anorexia, change in taste and early satiety, which were associated with malnutrition [25]. However, it is up to health professionals to understand that patients with multiple symptoms of nutritional impact, especially with chronic hypermetabolic and hypercatabolic diseases, are more susceptible to the malnutrition process and, consequently, have compromised quality of life.

In nephropathic patients, the identification of NIS is a nutritional screening tool that allows identification of the risk of malnutrition and has been associated with the length of stay in the hospital [26]. These results show that NIS are also common in other pathologies, which reinforces the importance of nutritional assessment to identify symptoms early and optimize nutritional therapy.

Anandavadivelan et al. [8], in a study using NIS combined with weight loss, found that the presence of three or more NIS has a significant impact on quality of life, and social and physical function 6 months after surgery for esophageal cancer. NIS contribute to the worsening of nutritional status and consequently involve a reduction in dietary intake, favoring catabolism, loss of strength, physical and mental fatigue [18].

The decrease in food consumption may occur due to the systemic effects of the disease, side effects of treatment or cytokine-mediated anorexia that may explain weight loss. Psychological factors such as fear, anxiety and depression can increase the complexity of the problem [22].

After regression analysis, the NIS that remained in the final model, increasing the risk of malnutrition, were anorexia, constipation and strange taste (moderate malnutrition), and constipation, mouth sores and others (severe malnutrition).

Constipation in the patient may be associated with occlusions of the GIT, due to neurological etiology or even with the high use of opioids, which lead to a slowdown in intestinal transit, reducing intestinal motility. Constipation is also associated with physical, social and emotional stress, abdominal pain, fatigue and hemorrhoids [27,28].

Kubrac et al. [29] evaluated the relationship between symptoms and reduced food intake in patients with head and neck cancer and found the symptoms anorexia, mouth sores, dysphagia and others as significant predictors of reduced oral food intake.

The significant association of the “other” NIS, which include depression and dental or financial problems, highlights the need to assess these symptoms in cancer patients. It is suggested that these factors affect the quality and quantity of food intake.

In this context, dietary counseling, the management of symptoms that impair dietary intake and the offer of oral nutritional supplements, if necessary, are recommended to ensure nutritional support in cancer patients who are at nutritional risk or have malnutrition [30].

The absence of some data, such as tumor staging and the absence/presence of metastasis, was considered a limitation of the study, as the information could help with a more effective nutritional diagnosis. Another limitation observed was the lack of studies that used PG-SGA to assess the NIS in cancer patients, which limited the discussion of these results.

## Conclusion

Malnutrition, the need for nutritional intervention and the presence of three or more NIS were elevated in the patients evaluated. NIS have been associated with worsening nutritional status, so their management and control should be considered in the care and treatment of cancer patients.

## Supporting information

S1 Data (SAV)
